# Dichotomous scoring of TDP-43 proteinopathy from specific brain regions in 27 academic research centers: associations with Alzheimer’s disease and cerebrovascular disease pathologies

**DOI:** 10.1186/s40478-018-0641-y

**Published:** 2018-12-19

**Authors:** Yuriko Katsumata, David W. Fardo, Walter A. Kukull, Peter T. Nelson

**Affiliations:** 1 Department of Biostatistics, University of Kentucky, 725 Rose Street, Lexington, KY 40536 USA; 2 Sanders-Brown Center on Aging, University of Kentucky, Lexington, KY 40536 USA; 3 National Alzheimer’s Coordinating Center, Department of Epidemiology, University of Washington, Seattle, WA 98105 USA; 4 Department of Pathology, Division of Neuropathology, University of Kentucky, Lexington, KY 40536 USA

**Keywords:** FTD, VCID, Arteriosclerosis, Apolipoprotein E

## Abstract

TAR-DNA binding protein 43 (TDP-43) proteinopathy is a common brain pathology in elderly persons, but much remains to be learned about this high-morbidity condition. Published stage-based systems for operationalizing disease severity rely on the involvement (presence/absence) of pathology in specific anatomic regions. To examine the comorbidities associated with TDP-43 pathology in aged individuals, we studied data from the National Alzheimer’s Coordinating Center (NACC) Neuropathology Data Set. Data were analyzed from 929 included subjects with available TDP-43 pathology information, sourced from 27 different American Alzheimer’s Disease Centers (ADCs). Cases with relatively unusual diseases including autopsy-proven frontotemporal lobar degeneration (FTLD-TDP or FTLD-tau) were excluded from the study. Our data provide new information about pathologic features that are and are not associated with TDP-43 pathologies in different brain areas—spinal cord, amygdala, hippocampus, entorhinal cortex/inferior temporal cortex, and frontal neocortex. Different research centers used cite-specific methods including different TDP-43 antibodies. TDP-43 pathology in at least one brain region was common (31.4%) but the pathology was rarely observed in spinal cord (1.8%) and also unusual in frontal cortex (5.3%). As expected, TDP-43 pathology was positively associated with comorbid hippocampal sclerosis pathology and with severe AD pathology. TDP-43 pathology was also associated with comorbid moderate-to-severe brain arteriolosclerosis. The association between TDP-43 pathology and brain arteriolosclerosis appears relatively specific since there was no detected association between TDP-43 pathology and microinfarcts, lacunar infarcts, large infarcts, cerebral amyloid angiopathy (CAA), or circle of Willis atherosclerosis. Together, these observations provide support for the hypothesis that many aged brains are affected by a TDP-43 proteinopathy that is more likely to be seen in brains with AD pathology, arteriolosclerosis pathology, or both.

**Electronic supplementary material:**

The online version of this article (10.1186/s40478-018-0641-y) contains supplementary material, which is available to authorized users.

## Introduction

There is an evolving appreciation of a common brain disease with TAR-DNA binding protein 43 (TDP-43) proteinopathy that mimics Alzheimer’s disease (AD) clinically [5, 25, 26, 34, 39, 50] and affects 10–25% of persons aged 85 or older [5, 19, 21, 33, 50]. The defining characteristics of this disease are recognized by neuropathologic observations: TDP-43 pathology, often with comorbid neuronal loss and astrocytosis pathology in the hippocampus [1, 33], the latter two features collectively termed hippocampal sclerosis (HS). The literature that pertains to this disease was initially focused on HS pathology (TDP-43 pathology was discovered as a disease marker in 2006 [38]), but it is now recognized that TDP-43 pathology is the most sensitive and specific marker of the disease. For example, cases with HS pathology due to acute anoxia is immunonegative for TDP-43 and is considered a fundamentally different disease [2, 20, 33]. Importantly, the presence of TDP-43 proteinopathy, with or without comorbid HS pathology, is independently associated with cognitive impairment [5, 26, 29, 31].

“TDP-43 pathology” lacks a universally applied specific connotation, but refers to phosphorylated TDP-43 deposits in cytoplasmic (where it may appear like speckles, skeins, or tangles), intranuclear, perivascular, and/or neurite-like structures. TDP-43 pathology may also manifest as a decrease in the normal (non-phosphorylated) TDP-43 in the nucleus [4], or within twig-like deposits of phosphorylated TDP-43 detected immunohistochemically in the subpial or subependymal regions [11, 18, 30]. In prior published papers that have studied the spectrum of TDP-43 pathologies in aged brains (often with comorbid AD pathology), the severity of TDP-43 proteinopathy has been mostly graded according to stage-based classification systems where the presence of any TDP-43 pathology in a given region defines a particular stage [15, 17, 27, 29, 44]. For example, the amygdala seems to be affected very early so this is the first stage. By contrast, in cases with extensive pathology, the frontal neocortex may be affected and if this region has any detectable TDP-43 pathology, that is indicative of a late disease stage. Unfortunately, there currently is no consensus as to a specific antibody or combination of antibodies recommended for detecting TDP-43 proteinopathy. Further, the stage-based classification systems for common age-related disease differ from TDP-43 pathologic staging systems that were developed for amyotrophic lateral sclerosis (ALS) and/or frontotemporal lobar degeneration (FTLD)-TDP [6, 10, 45].

Prior published findings suggest that vascular factors may cause or exacerbate the disease process that manifests neuropathologically as TDP-43 (with or without HS pathology) in the aged brain [8, 41, 47, 49]. In prior work, arteriolosclerosis – dysmorphic changes in small arterioles – was preferentially associated with this disease [36]. Further, arteriolosclerosis was observed in regions outside of the hippocampal formation in cases with comorbid HS pathology, indicating a “whole-brain disease” rather than a disease process isolated to the medial temporal lobe [37]. However, the precise underlying mechanisms are not understood, and more work is required to determine how the clinical and pathologic endpoints are associated with each other.

The AD Centers (ADCs) program has constituted a critical resource for research on AD and related dementias in the U.S. This network derived from a National Institutes of Health (NIH)-funded initiative that started in 1984 and has included more than 30 different ADCs geographically dispersed across the U.S. Each ADC follows a longitudinal cohort of generally elderly individuals reflecting a broad spectrum of clinical diseases and pathologic manifestations. The National Alzheimer’s Coordinating Center (NACC) oversees data collection by the ADCs. For research subjects that died and came to autopsy, a standardized form was created by NACC to describe the neuropathology in a systematic manner, and for correlation with clinical, radiographic, genetic, and biochemical parameters in the same persons. The latest Neuropathology (NP) Form was updated in 2014, and is referred to as version 10 (v10). The NACC NP Form v10 incorporated detailed neuropathological data including Thal phase for Aβ plaques [43], relatively  newly categorized FTLD neuropathologic changes [23], ALS/motor neuron disease (MND), HS of the CA1 and/or subiculum, and distributions of TDP-43 immunoreactive inclusions in five brain regions. The summary data for the updated v10 form was recently described [3]. Here we focused on the clinical and pathologic correlates of TDP-43 pathology in the NACC NP v10 data set among individuals lacking unusual conditions such as FTLD.

## Materials and methods

### Participants

For the current study, data (before exclusion criteria were applied) derived from 30 different ADCs with autopsies reported using the NACC NP v10 form, which started in 2014, through the data freeze of July 11th 2018. Autopsies were performed within each of the contributory ADCs. The database comprises a standardized set of data collected based on the NACC NP v10 data collection form (https://www.alz.washington.edu/NONMEMBER/NP/rdd_np.pdf). Inclusion criteria for this study were neuropathology data available through the NACC NP Form v10, age at death ≥65 years, and non-missing data on TDP-43 referent to at least one of the five brain regions of interest (see below). Exclusion criteria were the presence of at least one of 19 rare neurological diseases (see Additional file 1: Table S1). Research using the NACC database was approved by the University of Washington Institutional Review Board. Informed consent was obtained from all participants at the individual ADCs. The NACC data were de-identified.

### Measurements

#### Neuropathology data

**Fig. 1:**
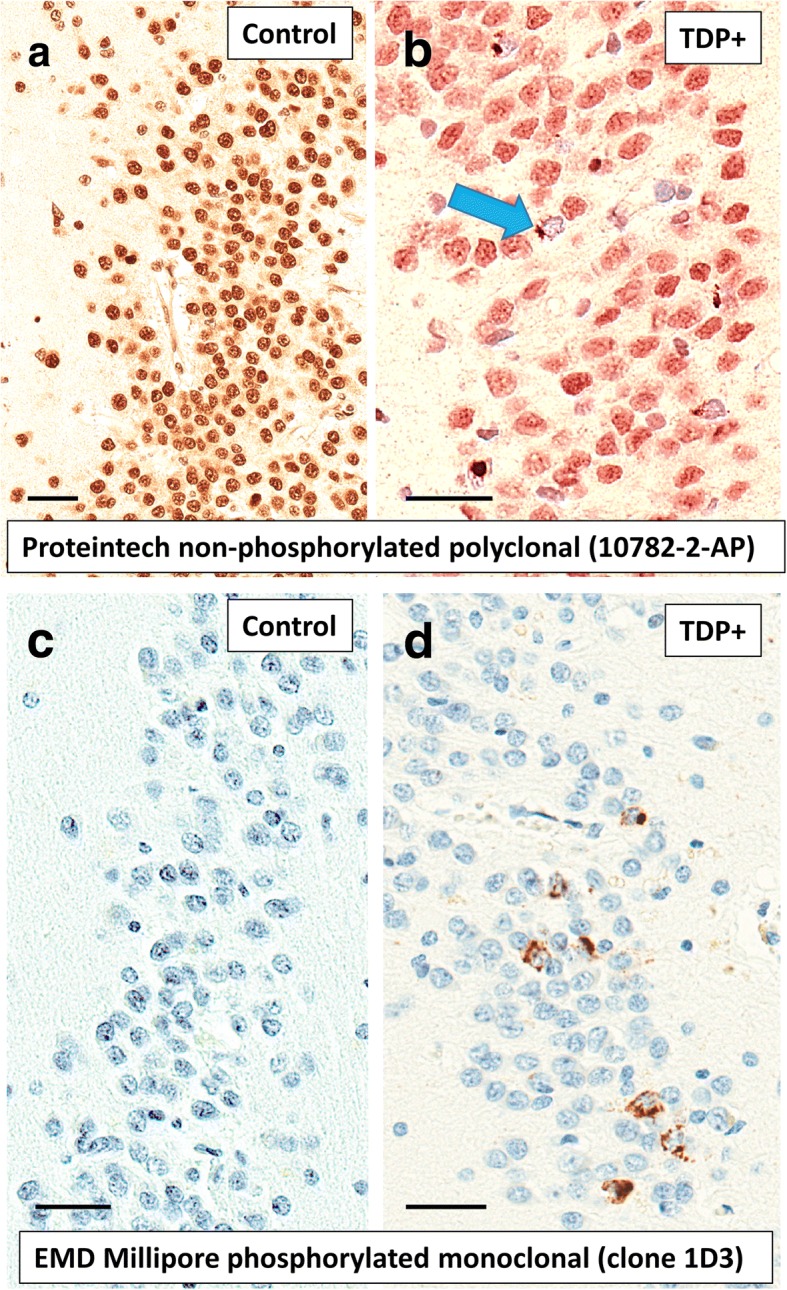
Examples of TDP-43 immunohistochemistry performed with the most commonly used antibodies against non-phosphorylated TDP-43 (**a**,**b**: Proteintech 10,782–2-AP), and phosphorylated TDP-43 (**c**,**d**: EMD Millipore clone 1D3) in the Alzheimer’s Disease Centers (ADCs) that contribute to the NACC neuropathology dataset. These sections are counterstained with hematoxylin (blue-stained nuclei). Shown are portions of dentate granule cells from the hippocampi of cases without TDP-43 pathology (**a**,**c**) which are contrasted against cases with detectable TDP-43 pathology (**b**,**d**). In normal brains, non-phosphorylated TDP-43 protein is seen in cell nuclei (**a**). In cases with TDP-43 pathology, the non-phosphorylated TDP-43 protein can be seen in some cells’ cytoplasm instead of the cell nucleus (**b**). The antibody against phosphorylated TDP-43 protein shows no brown reaction product in normal brain (**c**), with only the hematoxylin (blue) counterstain evident. By contrast, there are clearly intracellular aggregates (brown-colored chromagen) of phosphorylated TDP-43 protein in panel (**d**). Scale bars = 30 μm

**Fig. 2:**
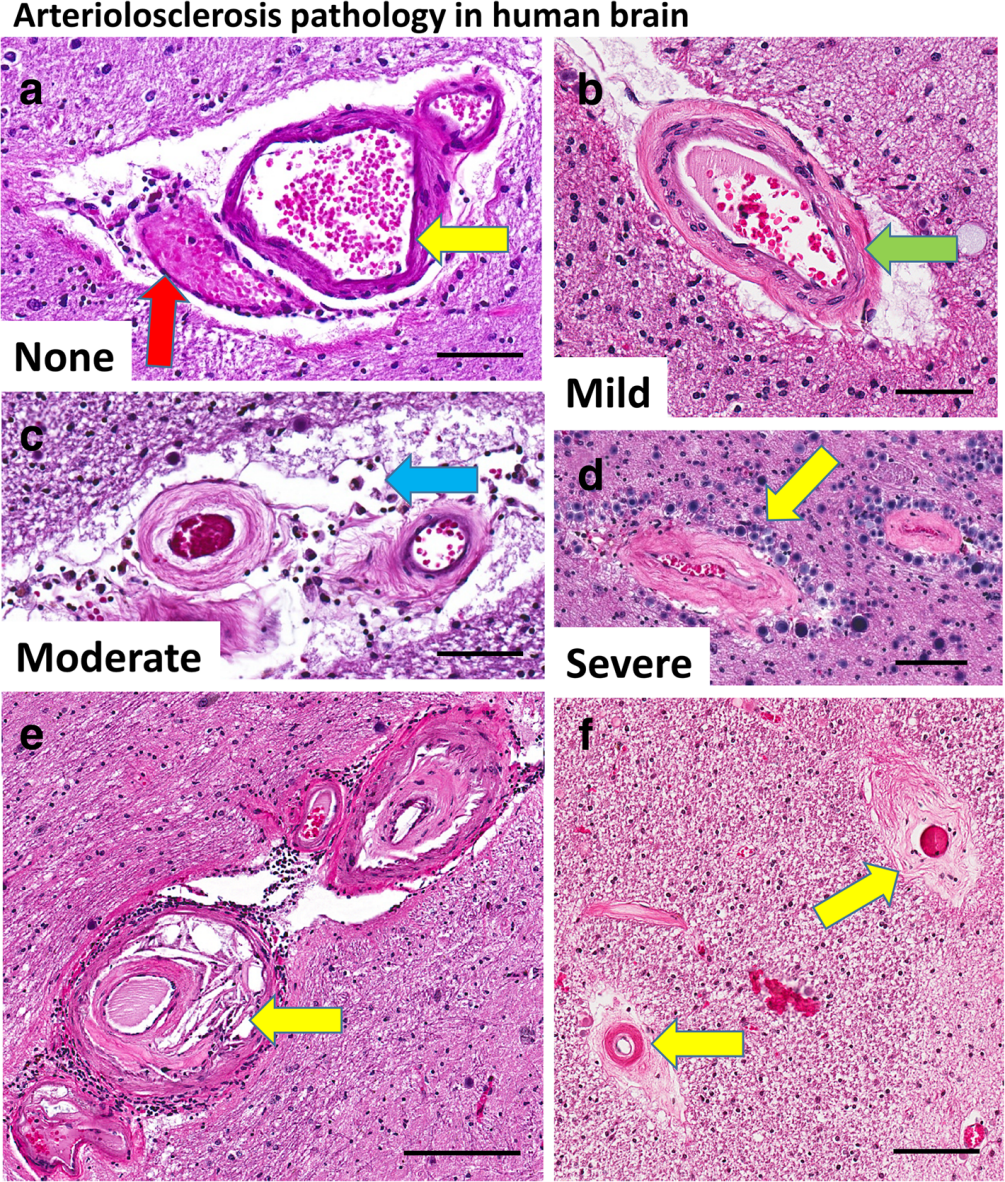
Arteriolosclerosis pathology in the aged human brain. The present study used a 0–3 scale of severity for arteriolosclerosis pathology, which is illustrated in panels (**a**-**d**). These photomicrographs depict vascular profiles in sections of amygdala or peri-amygdaloid regions from aged individuals at various stages of arteriolosclerosis pathology. Panel (**a**) shows an arteriole (yellow arrow) next to a presumed venule (red arrow), which have histopathologic features within normal limits in aging, including some eosinophilic material in the vessel wall and Virchow-Robin space [*] that may be partly an artifact of fixation and embedding. Panel (**b**) shows an arteriole with thicker vessel wall and intact cellular constituents, but with eosinophilic material (green arrow) in the adventitia. Panel (**c**) depicts arteriolosclerosis of moderate severity with some “onion-skinning” of the vessel wall and extravasation of macrophages (blue arrow) in the Virchow-Robin space. Panel (**d**) shows severe arteriolosclerosis in two vessels that have extensive proliferation of eosinophilic material in the vessel wall, attenuation of normal arteriolar cellular contents, apparent partial occlusion of the vessel itself, and the vessels are surrounded by corpora amylacea (yellow arrow) indicating localized brain injury. The pathologic appearance of brain arteriolosclerosis is heterogeneous, particularly in the area of the brain (amygdala; panels **e**,**f**) that is at high risk for incipient TDP-43 pathology. In panel (**e**), one can see pathologically affected blood vessel(s) with surrounding leukocytes and eosinophilic and small slit-like profiles that appear to be cholesterol clefts in the vessel wall. In panel (**f**), two vessels are seen (arrows) with largely attenuated cellular contents in the vessel walls. Scale bars = 150 um (**a**,**d**); 100 um (**b**,**c**), 300 um (**e**), 250 um (**f**)

TDP-43-immunoreactive inclusions were evaluated in five brain regions: spinal cord, amygdala, hippocampus, entorhinal cortex/inferior temporal cortex (EC/inferior TCTX), and frontal neocortex, with the response categories “no”, “yes”, “not assessed”, and “missing/unknown”. Because data on TDP-43 pathology in spinal cord contained more “not assessed’” or “missing/unknown” values (Additional file 1: Table S2), we considered TDP-43 inclusions in four brain regions (all except spinal cord) in the subsequent multivariable regression analyses. HS was determined by the variable of “hippocampal sclerosis of CA1 and/or subiculum” ("unilateral", "bilateral", or "present but laterality not assessed"). Data was obtained from all 30 contributory ADCs on whether the antibody used was phospho-specific or non-phospho-specific. A survey was sent to all the ADC Neuropathology Core leaders as to what specific antibodies were used. The phospho-specific antibodies were mostly 1D3 (EMD Millipore), followed by 11–9 (Cosmo Bio) and the polyclonal Cosmo Bio antibody (TIP-PTD-P02). The non-phospho-specific antibodies were almost all Proteintech 10,782–2-AP (rabbit polyclonal), with one center reporting to use Sigma C-term (T1580). Examples of results from the two most frequently used antibodies are shown in Fig. 1. For AD-related pathology, data were included on density of diffuse plaques ("none", "sparse", "moderate", or "frequent"), density of neocortical neuritic plaques ("none", "sparse", "moderate", or "frequent"), Thal phase for Aβ distribution (Thal Aβ phases 0 to 5), Braak stage for neurofibrillary degeneration (Braak NFT stages 0 to VI). For cerebrovascular pathology, data were available on atherosclerosis severity in the circle of Willis ("none", "mild", "moderate", or severe), cerebral amyloid angiopathy ("none", "mild", "moderate", or "severe"), infarct and lacunes (no or yes), microinfarcts (no or yes), hemorrhages and microbleeds (no or yes), and arteriolosclerosis ("none", "mild", "moderate", or "severe"; see Fig. 2). The detailed response categories of these variables and the dichotomized scoring are shown in Additional file 1: Table S3.

### Statistical analysis

Descriptive statistical analyses were performed for sex, age at death (both available in the NACC NP Form v10), and years of education, apolipoprotein E (*APOE*) genotype (no ε4 alleles = 0, one ε4 allele = 1, or pair of ε4 alleles = 2), and other health conditions at the last clinical visit (via self-report) including diabetes, hypertension, hypercholesterolemia, and thyroid disease (all from the NACC Uniform Data Set (UDS)).

Comparisons of characteristics of individuals with and without the TDP-43 pathology were performed using t-tests for continuous variables and Pearson’s chi-square test for categorical variables. Multivariable logistic regression was used to examine the associations of TDP-43 pathology with AD and cerebrovascular disease pathologies. We controlled for sex, age at death, *APOE* genotype, and the type of TDP-43 antibody in the analyses for AD pathologies, and additionally for Braak NFT stage and Thal Aβ phase in the analyses for cerebrovascular disease pathologies. All statistical analyses were carried out with R version 3.4.4 [40]. Statistical significance was set at 0.05.

## Results

**Fig. 3:**
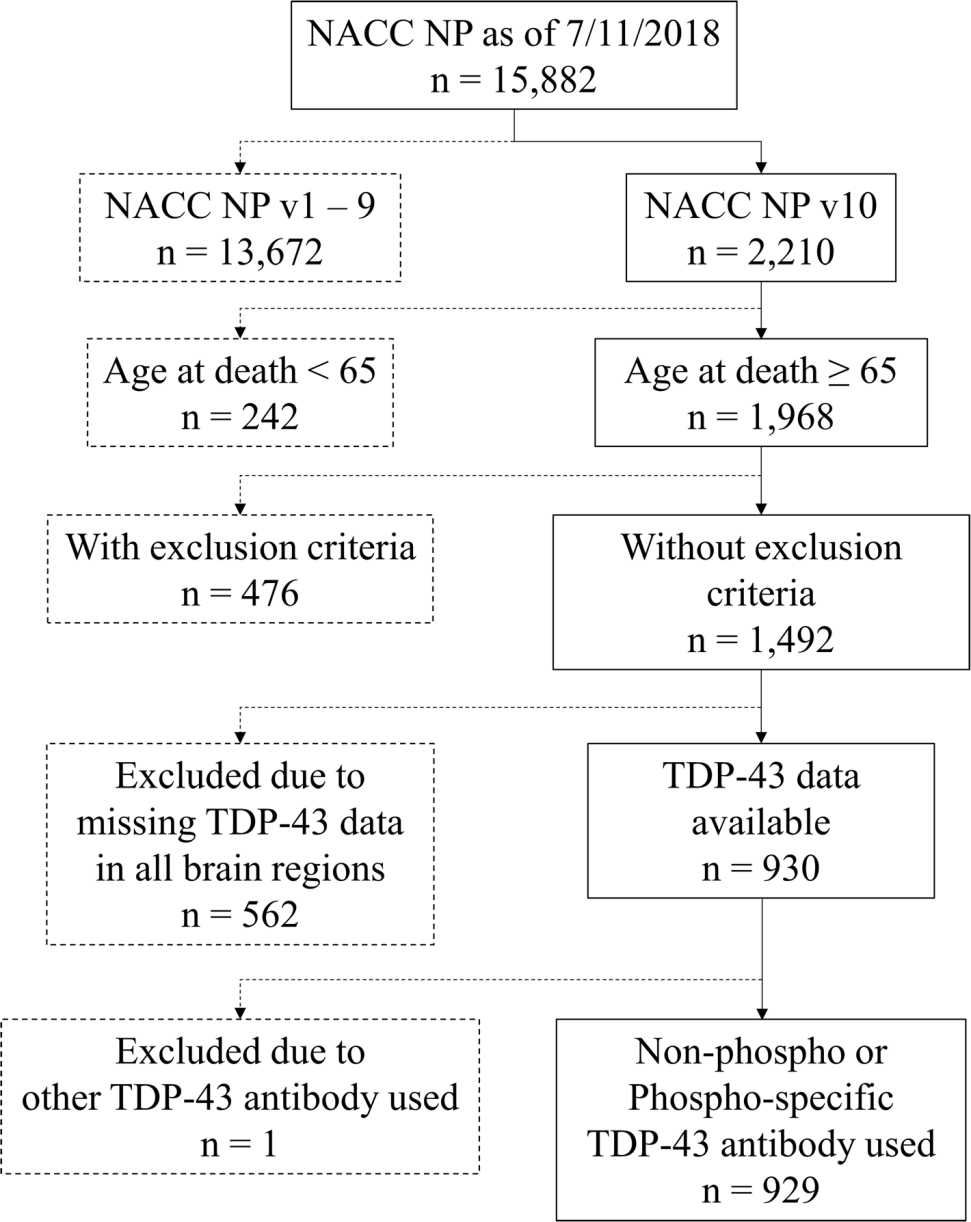
Included study subjects for the present study. NACC = National Alzheimer’s Coordinating Center; NP = Neuropathology

Subjects who were assessed by ADC neuropathologists using the NACC NP Form v10 and died at age 65 years or older (*n* = 1968) were extracted from the NACC NP dataset. We excluded 476 subjects who had at least one rare neurological disease listed in Additional file 1: Table S1, and we also excluded 562 subjects who had no TDP-43 pathology (i.e., missing) data reported in all five brain regions and 1 subject with “other” reported as the TDP-43 antibody used (Fig. 3). Following exclusions, a total of 929 subjects were included in this study. For these subjects, TDP-43 pathology data were sourced from 27 different ADCs (range of the number of subjects with any TDP-43 pathology in this study: 1–123 cases per center), including ADCs that only recently began to perform autopsies for inclusion in this dataset. The mean number of the subjects analyzed per ADC was 34 (median 33). Overall, 67.3% of the subjects were stained with phospho-specific antibodies; the rest with non-phospho-specfic antibodies. Among the 20 different ADCs that submitted 10 or more subjects with relevant TDP-43 immunohistochemistry (IHC) data to NACC, 13 ADCs used all or mostly phospho-specific antibodies for TDP-43 IHC, whereas 7 ADCs used all or mostly non-phospho-specific TDP-43 antibodies (Additional file 1: Table S4).

Table 1 shows the characteristics of the study subjects in total and stratified by TDP-43 pathology absent or present in at least one brain region. In all included subjects, the mean (standard deviation (SD)) age at death was 83.1 (8.7), 51.8% were males, and the mean of years of education was 15.5 (3.1). The subjects with TDP-43 pathology in at least one region died older (*p*-value < 0.001). There were no statistically significant differences in sex, *APOE* genotype, educational attainment, and other health conditions between subjects with and without TDP-43 pathology.

**Table 1:** Characteristics of included study subjects

Characteristic	All included subjects (*n* = 929)	No TDP-43 pathology (*n* = 637)	TDP-43 pathology at least one region^a^ (*n* = 292)	*P*-value^b^	Excluded subjects^c^ (*n* = 563)	*P*-value^d^
Age at death, mean ± SD	83.1 ± 8.7	82.4 ± 8.8	84.8 ± 8.5	**< 0.001**	84.4 ± 9.0	**0.012**
Gender, n (%)						
Male	481 (51.8)	341 (53.5)	140 (47.9)	0.13	287 (51.0)	0.81
Female	448 (48.2)	296 (46.5)	152 (52.1)		276 (49.0)	
Education (years), mean ± SD	15.5 ± 3.1	15.5 ± 3.1	15.5 ± 3.2	0.95	15.2 ± 3.3	0.10
*APOE*, n (%)						
−/−	370 (49.0)	275 (51.6)	95 (42.8)	0.063	229 (52.8)	0.43
−/ε4	309 (40.9)	210 (39.4)	99 (44.6)		162 (37.3)	
ε4/ε4	76 (10.1)	48 (9.0)	28 (12.6)		43 (9.9)	
Diabetes, n (%)						
Recent/remote	91 (15.5)	66 (15.6)	25 (15.2)	1	56 (13.2)	0.35
Absent	495 (84.5)	356 (84.4)	139 (84.8)		368 (86.8)	
Hypertension, n (%)						
Recent/remote	375 (64.3)	270 (64.1)	151 (64.8)	0.95	281 (67.9)	0.27
Absent	208 (35.7)	105 (35.9)	57 (35.2)		133 (32.1)	
Hypercholesterolemia, n (%)						
Recent/remote	411 (63.7)	300 (63.6)	111 (64.2)	0.96	297 (66.4)	0.39
Absent	234 (36.3)	172 (36.4)	62 (35.8)		150 (33.6)	
Thyroid disease present, n (%)						
Yes	70 (24.4)	48 (24.5)	22 (24.2)	1	31 (22.5)	0.75
No	217 (75.6)	148 (75.5)	69 (75.8)		107 (77.5)	

^a^TDP-43 pathology at least one of the five regions: spinal cord, amygdala, hippocampus, EC/inferior TCTX, and frontal neocortex

^b^The p-values were computed for the associations with TDP-43 inclusions statuses

^c^Subjects excluded due to missing TDP-43 data in all regions or other TDP-43 antibody used

^d^The *p*-values were computed for the associations between all included subjects and the subjects with no data on TDP-43 inclusions

*SD* standard deviation

Bold *p*-value represents the statistical significance

**Fig. 4:**
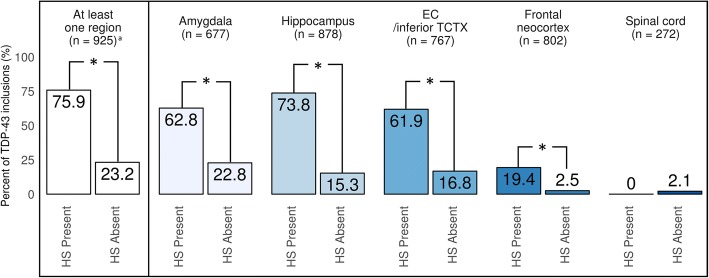
Comparisons in percent of TDP-43 pathology in each brain region between hippocampal sclerosis present and absent. Note that there is a strong association between hippocampal sclerosis (HS) pathology and TDP-43 pathology. However, only a minority of cases, with or without comorbid HS pathology, have TDP-43 pathology detected in frontal cortex or spinal cord. ^a^ *n* = 4 had missing data on hippocampal sclerosis (HS) pathology. * *p* < 0.001. EC = entorhinal cortex; TCTX = temporal cortex

We compared TDP-43 pathology frequencies between HS present and absent in each brain region (Fig. 4). HS pathology was strongly associated with TDP-43 pathology in each brain region except spinal cord. Table 2 shows the associations between TDP-43 and AD-related pathologies in each brain region. AD pathologies were strongly and positively associated with TDP-43 pathology in amygdala, hippocampus, and EC/inferior TCTX, although the association between dichotomized diffuse plaques (moderate/frequent vs. no/sparse) and TDP-43 pathology in hippocampus was not statistically significant. Among cerebrovascular disease pathologies, there were no significant associations (or even consistent trends) between TDP-43 pathology in any brain regions with atherosclerosis of the circle of Willis, cerebral amyloid angiopathy, infarct and lacunes, microinfarcts, hemorrhages or microbleeds.

**Table 2:** Associations between TDP-43 and Alzheimer’s disease pathologies using binary logistic regression (*n* = 929)

Region	OR (95% CI)^a^	*P*-value
Diffuse plaques (moderate + frequent vs. no + sparse)		
Amygdala	3.34 (1.47–8.99)	**0.0079**
Hippocampus	1.45 (0.81–2.71)	0.23
EC/inferior TCTX	2.61 (1.20–6.53)	**0.025**
Frontal neocortex	2.30 (0.63–14.93)	0.28
Neuritic plaques (moderate + frequent vs. no + sparse)		
Amygdala	2.84 (1.57–5.44)	**9.1 × 10** ^**− 4**^
Hippocampus	2.56 (1.58–4.29)	**2.2 × 10** ^**− 4**^
EC/inferior TCTX	4.04 (2.11–8.43)	**6.6 × 10** ^**− 5**^
Frontal neocortex	1.88 (0.71–5.92)	0.23
Thal Aβ phase (phase 4 + 5 vs. phase 0 to 3)		
Amygdala	2.78 (1.56–5.21)	**8.5 × 10** ^**− 4**^
Hippocampus	2.34 (1.44–3.93)	**8.5 × 10** ^**− 4**^
EC/inferior TCTX	2.52 (1.41–4.77)	**0.0029**
Frontal neocortex	1.42 (0.53–4.50)	0.51
Braak NFT stage (stage V + VI vs. stage 0 to IV)		
Amygdala	3.38 (1.99–5.95)	**1.3 × 10** ^**− 5**^
Hippocampus	2.90 (1.86–4.63)	**4.2 × 10** ^**− 6**^
EC/inferior TCTX	3.15 (1.86–5.53)	**3.4 × 10** ^**− 5**^
Frontal neocortex	1.22 (0.52–3.06)	0.66

^a^Odds ratios were adjusted for sex, age at death, *APOE* genotype, and the type of TDP-43 antibody

*OR* odds ratio, *CI* confidence interval, *EC* entorhinal cortex, *TCTX* temporal cortex, *NFT* neurofibrillary tangle

Bold *p*-value represents the statistical significance

By contrast, arteriolosclerosis pathology was associated with TDP-43 pathology in amygdala and EC/inferior TCTX when adjusted for sex, age at death, *APOE* genotype, and the type of TDP-43 antibody. The significant association in EC/inferior TCTX remained after AD-pathologies were added as covariates in the model (Table 3). To examine whether *APOE* genotype difference affects the association between TDP-43 pathology and arteriolosclerosis pathology, we further performed logistic regression analyses stratified by *APOE* genotype. As shown in Table 4, the associations of TDP-43 pathology in amygdala and EC/inferior TCTX with arteriolosclerosis pathology were observed in the subjects with *APOE* −/− or −/ε4 genotype. The significant association of TDP-43 in EC/inferior TCTX remained after including Thal Aβ phase and Braak NFT stage as additional covariates in the model. However, in persons with *APOE* ε4/ε4 (*n* = 77), TDP-43 pathologies in amygdala, hippocampus, and frontal neocortex were not associated with arteriolosclerosis pathology.

**Table 3:** Associations between TDP-43 and cerebrovascular disease pathologies using binary logistic regression (*n* = 929)

Region	Model 1^a^		Model 2^b^	
	OR (95% CI)	*P*-value	OR (95% CI)	*P*-value
Atherosclerosis of the circle of Willis (moderate + severe vs. none + mild)				
Amygdala	0.96 (0.63–1.47)	0.87	0.90 (0.58–1.39)	0.63
Hippocampus	1.07 (0.74–1.55)	0.70	0.98 (0.67–1.43)	0.93
EC/inferior TCTX	1.16 (0.77–1.75)	0.48	1.07 (0.70–1.62)	0.77
Frontal neocortex	1.03 (0.47–2.27)	0.93	1.00 (0.45–2.21)	0.99
Cerebral amyloid angiopathy (moderate + severe vs. none + mild)				
Amygdala	0.82 (0.52–1.28)	0.38	0.66 (0.41–1.04)	0.077
Hippocampus	0.89 (0.59–1.32)	0.57	0.71 (0.47–1.07)	0.10
EC/inferior TCTX	0.99 (0.64–1.52)	0.97	0.79 (0.50–1.22)	0.29
Frontal neocortex	1.16 (0.49–2.60)	0.73	1.11 (0.46–2.56)	0.81
Infarct and lacunes (yes vs. no)				
Amygdala	0.77 (0.43–1.32)	0.35	0.84 (0.47–1.45)	0.54
Hippocampus	0.76 (0.45–1.23)	0.28	0.81 (0.48–1.33)	0.42
EC/inferior TCTX	0.79 (0.45–1.32)	0.38	0.87 (0.49–1.48)	0.61
Frontal neocortex	1.64 (0.63–3.82)	0.27	1.66 (0.63–3.89)	0.26
Microinfarcts (yes vs. no)				
Amygdala	1.15 (0.73–1.79)	0.55	1.08 (0.68–1.70)	0.74
Hippocampus	1.14 (0.76–1.70)	0.51	1.14 (0.75–1.70)	0.54
EC/inferior TCTX	1.25 (0.80–1.94)	0.31	1.21 (0.77–1.88)	0.41
Frontal neocortex	1.76 (0.78–3.84)	0.16	1.77 (0.78–3.86)	0.16
Hemorrhages and microbleeds (yes vs. no)				
Amygdala	0.65 (0.26–1.44)	0.32	0.65 (0.25–1.47)	0.33
Hippocampus	0.71 (0.30–1.50)	0.40	0.69 (0.29–1.46)	0.36
EC/inferior TCTX	0.57 (0.21–1.29)	0.21	0.55 (0.20–1.26)	0.19
Frontal neocortex	0.45 (0.02–2.34)	0.45	0.44 (0.02–2.32)	0.44
Arteriolosclerosis (moderate + severe vs. none + mild)				
Amygdala	1.56 (1.03–2.37)	**0.038**	1.38 (0.90–2.13)	0.14
Hippocampus	1.31 (0.92–1.89)	0.14	1.16 (0.80–1.69)	0.42
EC/inferior TCTX	1.77 (1.17–2.72)	**0.0078**	1.61 (1.05–2.49)	**0.029**
Frontal neocortex	0.97 (0.45–2.13)	0.95	0.93 (0.43–2.06)	0.86

^a^Odds ratios were adjusted for sex, age at death, *APOE* genotype, and the type of TDP-43 antibody used

^b^Thal Aβ phase and Braak NFT stage were included as additional covariates in model 2

*OR* odds ratio, *CI* confidence interval, *EC* entorhinal cortex, *TCTX* temporal cortex

Bold *p*-value represents the statistical significance

**Table 4:** Associations between TDP-43 and arteriolosclerosis (moderate/severe vs. none/mild) pathologies among included subjects stratified by *APOE* genotype

Region	*APOE* −/− or −/ε4 (*n* = 679)^a^		*APOE* ε4/ε4 (*n* = 76)^a^	
	OR (95% CI)	*P*-value	OR (95% CI)	*P*-value
Model 1^b^				
Amygdala	1.66 (1.07–2.59)	**0.023**	0.71 (0.16–3.19)	0.65
Hippocampus	1.40 (0.96–2.05)	0.083	0.89 (0.29–2.79)	0.84
EC/inferior TCTX	1.82 (1.18–2.86)	**0.0080**	1.43 (0.35–6.55)	0.62
Frontal neocortex	1.13 (0.50–2.61)	0.76	0.26 (0.01–3.04)	0.30
Model 2^c^				
Amygdala	1.43 (0.91–2.26)	0.12	0.57 (0.12–2.67)	0.47
Hippocampus	1.19 (0.80–1.77)	0.39	0.80 (0.25–2.56)	0.70
EC/inferior TCTX	1.60 (1.02–2.54)	**0.041**	1.13 (0.27–5.26)	0.87
Frontal neocortex	1.08 (0.47–2.52)	0.86	0.21 (0.01–2.47)	0.23

^a^A total of 755 subjects had data on *APOE* genotype (those data were missing in 174 subjects)

^b^Odds ratios were adjusted for sex, age at death, and the type of TDP-43 antibody used

^c^Thal Aβ phase and Braak NFT stage were included as additional covariates in model 2

*OR* odds ratio, *CI* confidence interval, *EC* entorhinal cortex, *TCTX* temporal cortex

Bold *p*-value represents the statistical significance

## Discussion

Here we present data focusing on TDP-43 pathology in the aged human brain, using a large sample with autopsy confirmation, sourced from multiple high-quality research centers. Our data provide new information about comorbidities that are and are not apparently associated with TDP-43 pathologies in different brain regions. TDP-43 pathology is strongly associated with advanced AD and brain arteriolosclerosis pathologies.

There are some potential pitfalls in our study sample, as we have discussed previously [3]. Contributory ADC cohorts tend to be enriched for rare, genetic, early-onset, and “pure” subtypes of diseases, including AD and many other degenerative conditions. In particular, this sample may be biased toward individuals with a clinical syndrome that mimics AD. The NACC-contributory ADCs also tend to recruit (and achieve autopsy consent for) Caucasian/white individuals of relatively high socioeconomic status; thus, there are relatively few non-Caucasian individuals or those lacking formal education. Further, ADCs apply exclusion criteria that can limit the number of autopsied participants with mental illness, substance abuse, physical disability, or other prevalent conditions. There also are challenges in data interpretation related to the lack of methodologic standardization between the ADCs in terms of TDP-43 IHC methods. This problem will probably plague multi-center studies for some time since our study confirms that different state-of-the-art research centers use different reagents to operationalize TDP-43 proteinopathy (~ 2/3rd of ADCs use phospho-specific TDP-43 antibodies, whereas the remaining ADCs use antibodies that recognize non-phosphorylated epitopes). We also recognize the current lack of knowledge about underlying mechanisms is a limitation, and our study does not describe how the brain arteriolosclerosis pathology spatially relates to the TDP-43 proteinopathy.

Despite the challenges and potential pitfalls, there also are considerable strengths related to this use of the NACC NP data set. We note that despite the abovementioned sources of data variability, our study found evidence of strong associations between TDP-43 proteinopathy and other factors (age, HS pathology, AD pathology, and arteriolosclerosis pathology). These data were collected from individuals who died and came to autopsy over the past 4 years (NACC NP Form v10), providing both fresh data and relatively up-to-date clinical and pathological testing modalities. The study of nearly 1000 brains with *APOE* genotype and TDP-43 pathology status in multiple brain regions is unusual, and the statistical power it provides is important. Further, the derivation of data from 27 different research centers with expertise in research in AD and related dementias is a strength because the autopsy data may be considered more generalizable than the results from a single neuropathologist or small group of pathologists. For the foreseeable future, it seems unlikely that all research centers will agree on a single protocol for TDP-43 IHC, and we consider it a strength that the current study incorporates results from multiple research centers using site-specific protocols.

There are three main findings that we describe: TDP-43 pathology is strongly associated with advanced AD pathology; TDP-43 pathology is associated with increasingly severe arteriolosclerosis pathology (particularly in non *APOE* ɛ4/ɛ4 carriers); and age-related TDP-43 pathology is predominantly seen in the medial temporal cortex, uncommon in frontal neocortex, and very rare in spinal cord.

There is a relatively large extant literature on the relatively common comorbidity of TDP-43 pathology with AD, providing a compelling evidence that the pathologies co-occur whether or not they directly interact mechanistically [14, 16, 27, 30, 48]. Staging schema have been proposed to describe how TDP-43 pathology is distributed in brains with comorbid AD pathology [15, 17, 27]. Notably, in multiple cohorts of aged persons, TDP-43 pathology is more strongly linked to HS than *early* AD pathology [5, 7, 26, 28, 33]. However, within the amygdala of subjects with *advanced* AD, protein misfolding (tau, Aβ, α-synuclein, and TDP-43 pathologies alike) tends to occur [14, 16].

Compared with AD, the literature on the association between TDP-43 pathology and cerebrovascular is smaller, and overlaps with the paradigm of hypoxia/ischemia. As stated by Zarow et al. [49], “HS has long been hypothesized to result from ischemic-hypoxic insult to the brain. The CA1 sector is fed by small end-arterioles from the anterior choroidal and posterior cerebral arteries and is known to be susceptible to hypoxic injury” (with citation to Ref [9]). Others have also published data compatible with a link between HS pathology and cerebrovascular disease [22, 41, 46, 47, 49]. However, we have found in various data sets previous evidence of a relatively specific association between the type of HS that frequently is comborbid with TDP-43 pathology, and brain arteriolosclerosis [12, 35–37]. In the present study, the specificity of that association was underscored since no other subtype of cerebrovascular pathology was linked to TDP-43 pathology. There currently is no proven mechanistic explanation for this association. We note that factors that are conventionally associated with arteriolosclerosis, such as diabetes or hypertension, do not appear to be specifically associated with TDP-43 pathology. Intriguingly, Montagne and colleagues recently showed that subtle blood-brain barrier dysfunction and “leaky vessels” in the human hippocampus precede cognitive impairment in advanced aging [24]. Winkler et al. [42] reported that pericyte damage could contribute to cognitive impairment through disruption of the neurovascular unit, which may relate to TDP-43 proteinopathy, rather than AD. There also have been described some genetic risk factors that may help explain the link between brain arteriolosclerosis and TDP-43 pathology [32], but more work is required in this area. We speculate that there may be some reason that the TDP-43 pathology is usually confined to the medial temporal lobe of aged individuals, perhaps analogous to how primary age-related tauopathy [13], in the absence of comorbid Aβ plaques, tends not to progress beyond Braak NFT stage IV. Considering this analogy, there may be, in some of the brains, a disease-accelerating factor, analogous to Aβ, which promotes TDP-43 pathology outside of the medial temporal lobe.

## Additional file

Additional file 1: **Table S1.** Exclusion criteria in the National Alzheimer’s Coordination Center Neuropathology Form. **Table S2.** Missing frequency of TDP-43 pathology in each brain region collected on the National Alzheimer’s Coordination Center Neuropathology Form version 10 (*n* = 929). **Table S3.** Variables for Alzheimer’s disease and cerebrovascular disease pathologies in the National Alzheimer’s Coordination Center Neuropathology Form version 10. **Table S4.** Frequency of TDP-43 antibody used in each Alzheimer’s Disease Center. (PDF 181 kb)
