# Supplementary material for: Dichotomous scoring of TDP-43 proteinopathy from specific brain regions in 27 academic research centers: associations with Alzheimer’s disease and cerebrovascular disease pathologies
Source: Acta Neuropathol Commun. 2018 Dec 19;6:142. doi: 10.1186/s40478-018-0641-y (PMC6299605; doi:10.1186/s40478-018-0641-y)
Supplement: Supplementary file 1 — Table S1. Exclusion criteria in the National Alzheimer’s Coordination Center Neuropathology Form. Table S2. Missing frequency of TDP-43 pathology in each brain region collected on the National Alzheimer’s Coordination Center Neuropathology Form version 10 (n = 929). Table S3. Variables for Alzheimer’s disease and cerebrovascular disease pathologies in the National Alzheimer’s Coordination Center Neuropathology Form version 10. Table S4. Frequency of TDP-43 antibody used in each Alzheimer’s Disease Center. (PDF 181 kb) [file 40478_2018_641_MOESM1_ESM.pdf]

Table S1. Exclusion criteria in the National Alzheimer's Coordination Center Neuropathology Form

Exclusion criteria	Variable name
Down syndrome	NACCDOWN
Pigment-spheroid degeneration/NBIA	NPPDXA
Multiple system atrophy	NPPDXB
Trinucleotide disease (Huntington disease, SCA, other)	NPPDXD
Malformation of cortical development	NPPDXE
Metabolic/storage disorder of any type	NPPDXF
White matter disease, leukodystrophy	NPPDXG
White matter disease, multiple sclerosis or other demyelinating disease	NPPDXH
Contusion/traumatic brain injury of any type, acute	NPPDXI
Contusion/traumatic brain injury of any type, chronic	NPPDXJ
Neoplasm, primary	NPPDXK
Neoplasm, metastatic	NPPDXL
Infectious process of any type (encephalitis, abscess, etc.)	NPPDXM
Herniation, any site	NPPDXN
Prion disease	NACCPRIO
FTLD-tau	NPFTDTAU
ALS/motor neuron disease (MND)	NPALSMND
CADASIL	NPPATH10
FTLD with TDP-43 pathology (FTLD-TDP)	NPFTDTDP

Table S2. Missing frequency of TDP-43 pathology in each brain region collected on the National Alzheimer's Coordination Center Neuropathology Form version 10 (n = 929)

Region	n (%)		
	Yes	No	Not assessed or missing/unknown
Spinal cord	5 (0.5)	271 (29.2)	653 (70.3)
Amygdala	193 (20.8)	488 (52.5)	248 (26.7)
Hippocampus	218 (23.5)	664 (71.5)	47 (5.1)
EC/inferior TCTX	177 (19.1)	594 (63.9)	158 (17.0)
Frontal neocortex	43 (4.6)	763 (82.1)	123 (13.2)

EC = entorhinal cortex; TCTX = temporal cortex

Table S3. Variables for Alzheimer's disease and cerebrovascular disease pathologies in the National Alzheimer's Coordination Center Neuropathology Form version 10

Variable	Variable name	Response category	Dichotomized
AD pathology			
Density of diffuse plaques	NACCDIFF	No, sparse, moderate, frequent	0 = no + sparse<br>1 = moderate + frequent
Density of neocortical neuritic plaques	NACCNEUR	No, sparse, moderate, frequent	0 = no + sparse<br>1 = moderate + frequent
Thal Aβ phase	NPTHAL	Phase 0 to 5	0 = phase 0 to 3<br>1 = phase 4 + 5
Braak NFT stage	NACCBRAA	Stage 0 to VI	0 = stage 0 to IV<br>1 = stage V + VI
Cerebrovascular disease pathology			
Atherosclerosis of the circle of Willis	NACCAVAS	None, mild, moderate, severe	0 = none + mild<br>1 = moderate + severe
Cerebral amyloid angiopathy	NACCAMY	None, mild, moderate, severe	0 = none + mild<br>1 = moderate + severe
Infarct and lacunes	NACCINF	No, yes	0 = no<br>1 = yes
Microinfarcts	NACCMICR	No, yes	0 = no<br>1 = yes
Hemorrhages and microbleeds	NACCHEM	No, yes	0 = no<br>1 = yes
Arteriolosclerosis	NACCARTE	None, mild, moderate, severe	0 = none + mild<br>1 = moderate + severe

Table S4. Frequency of TDP-43 antibody used in each Alzheimer's Disease Center

ADC	TDP-43 antibody		Total
	Phospho-specific	Non-phospho-specific	
1	123	0	123
2	98	0	98
3	3	67	70
4	55	0	55
5	0	54	54
6	51	0	51
7	0	49	49
8	46	0	46
9	41	0	41
10	41	0	41
11	40	0	40
12	0	36	36
13	7	27	34
14	0	33	33
15	30	0	30
16	5	22	27
17	23	0	23
18	21	0	21
19	15	0	15
20	12	3	15
21	9	0	9
22	0	8	8
23	0	4	4
24	2	0	2
25	2	0	2
26	0	1	1
27	1	0	1
Total	625	304	929
